# Influence of Dextran Solution and Corneal Collagen Crosslinking on Corneal Biomechanical Parameters Evaluated by Corvis ST In Vitro

**DOI:** 10.3390/bioengineering11111156

**Published:** 2024-11-17

**Authors:** Xiao Qin, Bi Hu, Lili Guo, Haixia Zhang, Lin Li, Ying Jie, Lei Tian

**Affiliations:** 1 Peking Union Medical College Hospital, Beijing 100730, China; 2 Beijing Key Laboratory of Fundamental Research on Biomechanics in Clinical Application, Capital Medical University, Beijing 100069, China; 3 School of Biomedical Engineering, Capital Medical University, Beijing 100069, China; 4 Beijing Institute of Ophthalmology, Beijing Tongren Eye Center, Beijing Tongren Hospital, Capital Medical University, Beijing Ophthalmology & Visual Sciences Key Laboratory, Beijing 100730, China; 5 Department of Ophthalmology, Xuzhou First People’s Hospital, Xuzhou 221002, China

**Keywords:** dextran solution, corneal collagen crosslinking (CXL), corneal biomechanical properties, corneal visualization Scheimpflug technology (Corvis ST), intraocular pressure (IOP)

## Abstract

**Purpose:** To analyze the influence of dextran solution and corneal collagen crosslinking (CXL) on corneal biomechanical parameters in vitro, evaluated by Corneal Visualization Scheimpflug Technology (Corvis ST). **Materials and Methods:** Forty porcine eyes were included in this study. Twenty porcine eyes were instilled with dextran solution for 30 min (10 eyes in 2% dextran solution and 10 eyes in 20% dextran solution). CXL treatment was performed in 10 porcine eyes; the other 10 porcine eyes were regarded as the control group. Each eye was fixed on an experimental inflation platform to carry out Corvis measurements at different IOPs. Corneal biomechanical parameters were calculated based on Corvis measurement. Statistical analysis was used to analyze the influence of dextran solution and CXL on corneal biomechanical parameters based on Corvis parameters. **Results:** The corneal energy-absorbed area (*A*_absorbed_) decreased after being instilled with dextran solution under IOP of 15 mmHg (*p* < 0.001); the elastic modulus (*E*) of the cornea instilled with 20% dextran solution was significantly higher than that instilled with 2% dextran solution (*p* < 0.001), since it decreased after being instilled with 20% dextran solution (*p* = 0.030); the stiffness parameter at the first applanation (SP-A1) increased after CXL (*p* < 0.001). **Conclusions:** Both dextran solution and CXL can change corneal biomechanical properties; the concentration of dextran solution can influence the corneal biomechanical properties, which may, in turn, affect the effectiveness of CXL. SP-A1 may be used as an effective parameter for the evaluation of CXL.

## 1. Introduction

Corneal Visualization Scheimpflug Technology (Corvis ST) is one of the most commonly used devices for corneal biomechanical properties evaluation in the clinic. Corvis ST uses a Scheimpflug high-speed camera to rapidly acquire 140 cross-section images of the cornea with a horizontal diameter of 8.5 mm in a period of 30 seconds, thereby obtaining information about the deformation process of the cornea under constant air pulse pressure. Various parameters can be obtained through the corneal deformation process. Parameters provided by Corvis have shown their value in the preliminary diagnosis of corneal diseases such as keratoconus. These parameters are related to corneal biomechanics, intraocular pressure (IOP), and corneal geometrical parameters. Thus, these parameters are called corneal biomechanics-related parameters [1,2,3,4,5,6,7]. Based on the Corvis measurement, the Stiffness Parameter (SP-A1), corneal tangent stiffness coefficient (*S*_TSC_) [8], corneal energy-absorbed area (*A*_absorbed_) [8], and the corneal elastic modulus (*E*) [9,10] have been calculated.

The corneal tangent stiffness coefficient is a parameter used to evaluate corneal elasticity, while the corneal energy-absorbed area is used to assess corneal viscosity. These two parameters effectively provide quantitative indicators of elasticity and viscosity and demonstrate excellent repeatability, showing significant clinical application value. Compared to traditional parameters, the corneal tangent stiffness coefficient and the corneal energy-absorbed area offer a more accurate and comprehensive method for evaluating corneal biomechanical properties. These parameters not only precisely identify corneal elasticity and viscosity but also exhibit superior predictive performance over existing parameters, particularly in the early diagnosis of keratoconus. However, the accuracy of these parameters is also affected by changes in video image quality and air puff force [8].

These parameters made it possible to obtain corneal biomechanical properties based on simple clinical measurements. These parameters have shown significant differences between normal and keratoconus corneas [8]. However, it is not known whether there are changes in the corneal tangent stiffness coefficient, corneal energy-absorbed area, and the corneal elastic modulus after clinical treatments such as CXL or dextran treatment in vivo or in vitro.

Corneal collagen crosslinking (CXL) has been considered an effective treatment approach that can halt or delay the progression of keratoconus [11,12,13,14,15]. CXL can not only enhance the overall structural stability of the cornea, but can also significantly enhance the defense capability of the corneal stroma. This enhanced defense makes the cornea more resistant to digestive enzymes. Therefore, CXL can be applied to the treatment of keratoconus, corneal ulcers, and other eye diseases, providing patients with a more effective treatment choice. The implementation of this technology helps to accelerate the healing process of the cornea, thus effectively improving the quality of vision and quality of life of patients. Conventional CXL is considered safe and effective for the prevention of keratoconus progression. It uses the UVX 1000 system(Peschke GmbH, Hünenberg, Switzerland) with 0.1% riboflavin solution presoaked in 20% dextran for 20 min and 3 mW/cm^2^ ultraviolet A (UVA) light for 30 min [16,17,18,19,20,21,22]. In this procedure, riboflavin interacts with UVA radiation to promote the production of reactive oxygen species, which forms covalent bonds of collagen molecules and promotes the recovery of corneal strength [20]. Under ultraviolet irradiation, riboflavin undergoes photodegradation to lumichromes and lumiflavin. This process results in a decrease in riboflavin concentration in the corneal stroma. Therefore, riboflavin must be supplemented during irradiation. A 20% dextran solution was used to maintain the solution iso-osmolar with respect to the corneal stroma [23]. In the treatment of keratoconus patients with a thin cornea, hypotonic dextran may be used to increase the corneal thickness by swelling the corneas in CXL [24,25,26,27,28,29]. Nevertheless, previous studies have found that the dextran solution is a vital component in the CXL treatment. Hypertonic CXL treatment results in a stiffness decrease [30], and the substitution of dextran with dextran sulfate in riboflavin solutions may result in the loss of vision and permanent corneal opacity [31].

Numerous studies that used in vitro experiments have reported variations in corneal biomechanical properties after CXL [32,33,34,35,36], showing that corneal stiffness increased after CXL. Some researchers have reported on the variation of corneal biomechanical parameters provided by Corvis; Steinberg et al. found that the first applanation time (A1T) increased and the second applanation time (A2T) decreased 3 months after CXL [37]. Sedaghat et al. found significant changes in radius at the highest concavity and an integrated inverse radius four years after CXL [38]. Xanthopoulou et al. found that SP-A1 in keratoconus patients two years after CXL was significantly higher than that of keratoconus patients who did not undergo CXL [39]. These studies have not reached an agreement in relation to the variation of corneal viscoelastic properties after CXL. To the best of our knowledge, only a few studies focused on the influence of dextran solution on corneal biomechanical properties. Elucidating the influence of dextran solution and riboflavin-ultraviolet CXL on corneal biomechanical properties may be helpful for the further cognition of corneal biomechanical properties as determined by Corvis. These will provide a theoretical basis for the individual design and prognosis of CXL.

In this study, porcine eyes were used as research objects, and corneal biomechanical parameters were determined based on Corvis ST [40]. This study has conducted biomechanical parameter testing on porcine corneas instilled with different concentrations of dextran solution, as well as on porcine corneas after CXL. Dextran is a polysaccharide extracted from natural glucose by bacteria. In ophthalmology, dextran is used as an eye lubricant. It increases the viscosity of the eye surface, helps retain tear fluid, and relieves dry eye symptoms [41]. It is also a commonly used material for performing CXL. In this study, each porcine eye was measured with Corvis ST under several different IOPs to evaluate the influence of IOP on the variation of corneal biomechanical parameters after dextran immersion or CXL. The results of this study will provide a theoretical basis for the design and prognosis of CXL.

## 2. Materials and Methods

### 2.1. Materials and Measurements

Forty porcine eyes were enrolled in this study. Porcine eyeballs were acquired from a local slaughterhouse and maintained in 0.9% normal saline at 4 °C before testing. The porcine eyes were randomly divided into four groups. Group 1 (2% dextran group) and Group 2 (20% dextran group) only used dextran solution for instilling porcine corneas. Group 1 used 2% dextran solution for corneal instillation every 3 min during the experiment for 30 min. Group 2 used 20% dextran solution for corneal instillation every three minutes during the experiment for 30 min. Group 3 (CXL group) underwent corneal collagen crosslinking. Conventional corneal collagen crosslinking consists of two steps: the first involves the instillation of a 0.1% riboflavin–20% dextran solution on the cornea, and the second involves UVA irradiation. Group 4 is the control group, which does not receive any treatment. Before the experiments, the whole epithelium was removed from the cornea. The determination of the sample size was grounded in power analysis, informed by the sample sizes utilized in analogous studies within the field.

The procedure of conventional CXL is as follows: Prior to irradiation, riboflavin 0.1%–dextran 20% solution (Ricrolin Sooft Italia, Montegiorgio, Fermo, Italy) was instilled on the central cornea every 3 min for 30 min. Next, the cornea was exposed to UVA emission for 30 min (370 nm, 3 mW/cm^2^); at the same time, the riboflavin 0.1%–dextran 20% solution was instilled every 3 min.

**Figure 1:**
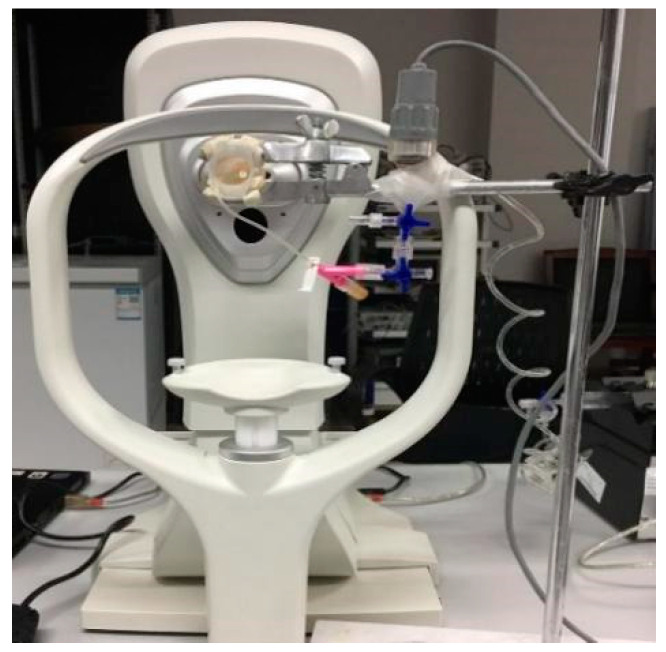
A platform for eyeball inflation and Corvis test.

Before the Corvis measurements, the corneal central thickness was measured with a Pachymeter SP-3000 (TOMEY, Aichi, Japan) three times. Because this experiment was in vitro, it was necessary to observe the cornea status. When comparing the values of the corneal biomechanical parameters between the different groups, the geometric factors of the cornea should be excluded, so the corneal thickness should be consistent during the experiment. In this experiment, corneal dehydration caused a change in corneal thickness. After that, every porcine eyeball was fixed on the self-built experimental inflation platform, as shown in Figure 1. Corvis measurements were carried out when the IOPs were stable at 15, 20, 25, and 30 mmHg. During the experiment, a 0.5 mm radius indwelling needle from the optic nerve was inserted into the anterior chamber to adjust the intraocular pressure. The pressure sensor and Corvis ST were connected to a computer to monitor intraocular pressure (IOP). IOP measurements were taken using the Pclab biomedical signal acquisition and processing system (Beijing Microsignalstar Technology Development Co., Ltd, Beijing, China). To adjust intraocular pressure, physiological saline was injected into the anterior chamber at a rate of 20 μL/min to control the IOP. Corvis tests were carried out when the IOPs were stable at 15, 20, 25, and 30 mmHg. All Corvis measurement tests were controlled within 5–8 min, and no significant dehydration was found. A study has shown that corneal dehydration can lead to changes in thickness, so the corneal thickness should be measured during the experiment to prevent corneal dehydration [42]. The tests were taken by the same technician and captured by automatic release to ensure the absence of user dependency. Corvis results on “Alignment” and “Pressure Profile” reading “OK” were accepted; otherwise, measurement was repeated until the reading was “OK”.

All the specimens were tested within no more than 24 h after death. All of the experiments were approved by the ethics committee of Capital Medical University. The experiments were performed in accordance with the ARRIVE guidelines and NIH guidelines.

### 2.2. Determining Corneal Biomechanical Parameters

**Figure 2:**
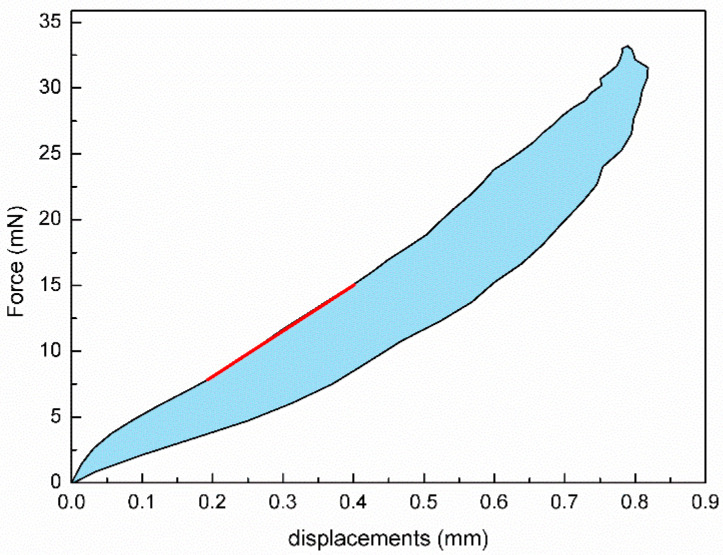
Air puff force–corneal apical displacement curve. The blue area is the energy-absorbed area. The red line stands for the tangent stiffness coefficient.

In addition to parameters provided by Corvis, the corneal tangent stiffness coefficient (*S*_TSC_), corneal energy-absorbed area (*A*_absorbed_), and corneal elastic modulus (*E*) were also determined based on the air puff forces–corneal apical displacement curve provided by Corvis following the methods reported in our previous studies [8,9]. The corneal energy-absorbed area and corneal tangent stiffness coefficient are based on the air puff force and corneal displacement measurement to characterize corneal elasticity and viscosity. The typical air puff forces–corneal apical displacement curve is shown in Figure 2. A specific fitting line was selected on the loading curve to represent the tangent stiffness coefficient (*S*_TSC_). The area between the loading and unloading curves was defined as the energy-absorbed area (*A*_absorbed_). Corneal viscoelastic behavior can be exhibited from the loading–unloading response. When Corvis measurements were regarded as indentation experiments and the cornea as a shallow spherical shell, the corneal elastic modulus was determined based on the relation between force and spherical apical displacement.

### 2.3. Statistical Analysis

The Kolmogorov–Smirnov test was used to check for a normal distribution of quantitative data, which were reported as the mean and standard deviation (SD). The coefficient of variation (CV) was calculated to evaluate the repeatability of corneal biomechanical parameters. The influence of dextran solution, CXL, and IOP on corneal biomechanical parameters was evaluated by multivariate analysis of variance (ANOVA). Bonferroni post hoc analysis was used to evaluate the significance of specific differences between groups. All statistical analyses were performed using SPSS statistical software version 21.0 (SPSS Inc., Chicago, IL, USA) and an alpha value of *p* < 0.05 was considered statistically significant.

## 3. Results

The Kolmogorov–Smirnov test results showed that all of the corneal Corvis parameters had a normal distribution (*p* > 0.2). The coefficient of variation of Corvis parameters related to corneal biomechanics was no more than 10%, which suggested that these parameters had good repeatability.

The porcine central corneal thickness (CCT) measured by the Pachymeter SP-3000 (TOMEY, Aichi, Japan) showed no significant difference among different groups before CXL or dextran solution immersion (*p* > 0.05).

The CCT and corneal radius (*R*) of the different groups after treatment are shown in Table 1. The results showed no significant difference among other groups (*p* > 0.05) in *R*. The CCT significantly increased after being soaked in 2% dextran solution (*p* < 0.001) and was significantly reduced after being soaked in 20% dextran solution (*p* < 0.001). There was no significant variation in CCT and *R* after CXL (*p* > 0.05).

**Figure 3:**
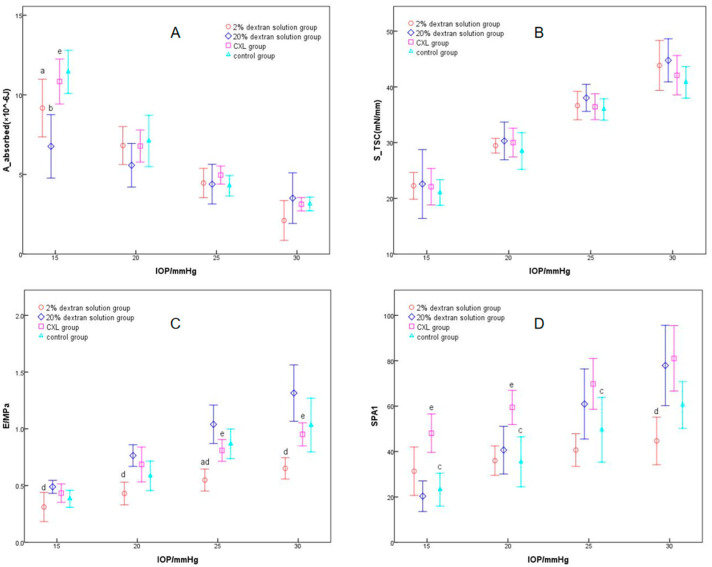
Variation of corneal biomechanical parameters with IOP in different groups (The graphs (**A**–**D**) represent the parameter changes in corneal energy-absorbed area (*A*_absorbed_), corneal tangent stiffness coefficient (*S*_TSC_), the corneal elastic modulus (*E*) and the Stiffness Parameter (SP-A1), respectively). (Statistical significance (*p <* 0.05) annotation: a, 2% dextran solution group and control group; b, 20% dextran solution group and control group; c, CXL group and control group; d, 2% dextran solution group and 20% dextran solution group; e, 20% dextran solution group and CXL group).

Figure 3 presents the corneal biomechanical parameters in different groups under different IOPs. *A*_absorbed_ decreased after being soaked in 2% and 20% dextran solution under an IOP of 15 mmHg (*p* < 0.001), and no significant difference was found at other IOPs (*p* > 0.05). STSC showed no significant difference among various groups *(p >* 0.05); *E* in 20% dextran solution group is significantly higher than that in 2% dextran solution group *(p <* 0.001), and it decreased after being instilled with 2% dextran solution and increased after being soaked in 20% dextran solution (*p* = 0.030). SP-A1 showed no significant difference after being soaked in 2% dextran solution and 20% dextran solution (*p >* 0.05). Compared to the CXL group and the control group in Figure 3, SP-A1 increased after corneal collagen crosslinking *(p <* 0.001), and there was no significant variation in other parameters after CXL (*p >* 0.05). In all of the four groups, SP-A1, *S*_TSC_, and *E* increased with IOP (*p <* 0.001), while *A*_absorbed_ decreased with IOP (*p <* 0.001). The results of the multivariate analysis of variance showed no interaction among these three factors in the four parameters above (*p >* 0.05).

## 4. Discussion

This paper evaluated the in vitro influence of dextran solution and CXL on corneal biomechanical parameters determined from Corvis measurements. The results showed that both dextran solution and CXL had an influence on corneal biomechanical properties. The results of this study may provide an important theoretical basis for the design and prognosis of CXL.

As an essential refractive medium, the cornea accounts for approximately three-quarters of refractive power. Consequently, maintaining the normal shape and structure of the cornea is essential for maintaining clear vision. The biomechanical properties of the cornea play a key role in maintaining its normal function.

Keratoconus is a progressive ectatic, non-inflammatory corneal disease that often occurs in adolescents. This disease is one of the main indications for corneal transplantation, which causes the local area of the cornea to decrease in biomechanical strength, which in turn causes the cornea to protrusion forward in a cone shape, resulting in irregular astigmatism and possibly vision loss. Corneal crosslinking surgery is an effective treatment method to enhance the corneal strength. Its principle involves instilling riboflavin solution into the cornea, combined with ultraviolet irradiation, to alter the biomechanical properties of the corneal collagen, thereby achieving corneal strengthening and preventing disease progression.

Numerous studies have reported on the corneal biomechanical properties after CXL [43,44,45,46] whilst failing to reach consensus on the variations of corneal biomechanical parameters after CXL. Dias et al. assessed the corneal anterior and posterior stromal elasticity after CXL using Atomic Force Microscopy (AFM) [32], showing that the stiffness of corneal anterior stroma increased after CXL, while the posterior stroma was not affected by CXL. Zhang and colleagues studied the corneal biomechanical properties after CXL with a uniaxial tensile test [35], and they found that corneal elastic moduli significantly increased after CXL. Matteoli et al. measured porcine corneal biomechanical properties with corneal inflation experiments [34] and found that the corneal elastic modulus increased after CXL under high IOP, while no significant difference was found within the physiological range of IOP (15–30 mmHg). Although not statistically significant, the elastic modulus slightly increased after CXL under an IOP of 15 mmHg and 20 mmHg. The lack of a significant increase in the corneal elastic modulus after CXL may be related to the in vitro CXL without the corneal remodeling process.

The clinical results showed that SP-A1 increased significantly 6 months after CXL [47], and no significant changes were found 4 years after CXL [38]. In this study, SP-A1 increased immediately after CXL, which was in agreement with the reported increase in corneal elastic properties from a short-term evaluation in vivo after CXL. In the current study, no significant changes were found in *S*_TSC_ and the corneal elastic modulus after CXL, which may be attributed to the CXL surgery being carried out in vitro and the corneal elastic modulus being measured under a physiological range of IOP [34]. Liu et al. found corneal viscosity decreased significantly after CXL via stress relaxation experiments [48]. Although *A*_absorbed_ was found, no significant variations were found after CXL in this study. This difference may be due to the 30 m duration of the air puff test, which was too short to achieve thermodynamic equilibrium [49].

The composition of riboflavin solution can influence the evaluation of the potential efficacy of CXL [13]. Riboflavin can be mixed with different solutions to achieve various effects. Research has shown that the combination of riboflavin with dextran solution effectively prevents excessive swelling of the corneal stroma. Furthermore, studies indicate that substituting hydroxymethyl cellulose (HPMC) for dextran in the riboflavin mixture can significantly accelerate the penetration rate of the solution into the corneal stroma, demonstrating higher penetration efficiency compared to the riboflavin and dextran mixture [50]. A 20% dextran solution is used in standard CXL to maintain the solution iso-osmolar with the corneal stroma [23]. However, for keratoconus patients with thin corneas (<400 μm), hypotonic dextran may be used to increase corneal thickness during CXL [24,26]. To the best of our knowledge, only a few studies have reported on the influence of dextran solution on corneal biomechanical properties.

In this study, it was found that the corneal viscoelastic parameters, *A*_absorbed_, decreased after being soaked in both 2% and 20% dextran solution under IOP of 15 mmHg. Corneal elastic modulus, *E*, increased after it was instilled with 20% dextran solution and decreased after it was instilled with 2% dextran solution. The influence of dextran solution on corneal biomechanical properties may be reflected in two aspects: the influence of dextran on the corneal structure [51] and the different concentration of dextran solution, which may lead to corneal swelling or shrinking [52,53,54]. Sondergaard et al. compared the corneal shear moduli in riboflavin solution-treated groups and CXL-treated groups, and the results showed there was no significant difference between these groups. This reveals that the immediate effects of CXL treatment may be due partly to the interaction between the ground substance and riboflavin–dextran solution [55]. In Kling’s study, dextran solution has been used to maintain the native corneal ultrastructure in corneal decellularization [56]. In Hatami-Marbini’s study, the corneal elastic modulus decreased with the increase in corneal swelling [57]. These results suggested that dextran may have an influence on corneal viscoelastic properties. The concentration of dextran may influence the corneal biomechanical because of the corneal swelling or corneal shrinkage.

Another parameter that can reflect corneal stiffness, SP-A1, was found to decrease after being instilled with 20% dextran solution and increased after being instilled with 2% dextran solution under an IOP of 15 mmHg, which was opposite to the variation in *E,* while SP-A1 showed a similar variation trend with *E* under other IOPs. These results may be because SP-A1 was more influenced by corneal thickness under an IOP of 15 mmHg. In our previous study, it was also found that *E* was less influenced by corneal geometrical parameters than SP-A1 [9].

In the current study, the influence of IOP on the corneal biomechanical parameters was also examined. Our results showed that SP-A1, *S*_TSC_, and *E* increased with IOP, and *A*_absorbed_ decreased with IOP. Furthermore, *A*_absorbed_ decreased after being soaked in dextran solution under an IOP of 15 mmHg, while no significant difference was found at other IOPs. These results suggest that the influence of IOP should be considered when evaluating corneal biomechanical parameters using Corvis measurements.

Comparing the four parameters addressed in this study, it was found that both dextran solution and CXL had an influence on the corneal biomechanical parameters. *E* was more sensitive to corneal swelling induced by dextran solution, and SP-A1 was more sensitive to corneal thickness variation induced by corneal swelling. The variation of SP-A1 was also more significant than *E* after CXL. According to these results, SP-A1 may be a useful parameter to evaluate the effect of CXL. Combining *E*, SP-A1, and *A*_absorbed_ can help ophthalmologists to design CXL procedures individually and to predict the effect of CXL whilst considering the influence of dextran solution and CXL, especially for the CXL treatment with a thin cornea.

The innovation of this study was that it evaluated the influence of CXL and dextran solution on corneal biomechanical properties using Corvis measurements. To the authors’ acknowledge, there are few studies that have studied the influence of dextran solution on corneal biomechanical properties using Corvis measurements. The results of this study showed that both dextran solution and CXL can change corneal biomechanical properties; the corneal elastic modulus (*E*) was more affected by the corneal swelling induced by the dextran solution, while SP-A1 was more influenced by the variation of corneal thickness induced by the dextran solution. SP-A1 may be used as an effective parameter for the evaluation of CXL. Dextran may influence the corneal viscoelastic properties. These results may provide a theoretical basis for the individualized design of CXL. This study has some limitations that need to be pointed out. First, only two concentrations of dextran solution were used to study the influence of the dextran solution on corneal biomechanical properties. Second, standard CXL was carried out in this study, during which the UV irradiation intensity and time were fixed. The results of this study revealed a significant difference between the biomechanical parameters of corneas soaked in 2% and 20% dextran solution. The variation of corneal biomechanical parameters after standard UVA riboflavin CXL was also found in this study. Future studies should use more concentrations of dextran solution, more gradients of the UVA irradiation intensity, and a greater amount of time to achieve better clinical application.

## Figures and Tables

**Table 1:** Porcine central corneal thickness and curvature radius in different groups.

	2% Dextran Group	20% Dextran Group	CXL Group	Control Group	*p*
**CCT/** **μ** **m**	983 ± 16	747 ± 21	877 ± 20	860 ± 22	<0.001
**R/mm**	8.10 ± 0.87	8.65 ± 1.30	8.54 ± 1.07	8.16 ± 1.67	0.154

## Data Availability

The original contributions presented in the study are included in the article; further inquiries can be directed to the corresponding authors.
